# Associations Between Cognitive Function, Balance, and Mobility in Community-Dwelling Older Adults With COPD

**DOI:** 10.1093/geroni/igaa057.741

**Published:** 2020-12-16

**Authors:** Shweta Gore, Jennifer Blackwood, Tyler Ziccardi

**Affiliations:** 1 MGH Institute of Health Professions, Charlestown, Massachusetts, United States; 2 University of Michigan-Flint, Flint, Michigan, United States

## Abstract

Older adults with chronic obstructive pulmonary disease (COPD) are at risk for physical and cognitive impairment. Cognitive function is associated with falls in older adults however it is unknown if a relationship exists between cognitive function and falls in COPD. The aim of this study was to examine the relationships between cognitive function and balance and mobility in older adults with COPD. A secondary analysis was performed using data from the 2010 wave of the Health and Retirement Study (HRS) (N=4051). Cognitive (immediate and delayed recall, executive function) and physical (gait speed, tandem balance time) measure data was extracted from older adults with COPD (N=382) and an age matched control group without COPD (N=382) who met inclusion/exclusion criteria. Multivariate linear regression modeling was performed to examine associations between cognitive function and mobility or balance while controlling for age, gender, BMI, grip strength, and education. We found that in COPD, immediate word recall, delayed word recall, orientation, and executive function (β ranging from 0.004-0.02) were significantly associated with gait speed while only delayed word recall (β = 0.122, p < .05) was associated with tandem balance. These same associations did not exist in those without COPD. In older adults with COPD, cognitive function is associated with balance and mobility. Screening for cognitive function, specifically delayed recall, should be a part of the management of falls in this population.

